# Incidence of type II diabetes in chronic obstructive pulmonary disease: a nested case–control study

**DOI:** 10.1038/s41533-019-0138-6

**Published:** 2019-07-15

**Authors:** Alicia Gayle, Scott Dickinson, Chris Poole, Marie Pang, Ornella Fauconnot, Jennifer K. Quint

**Affiliations:** 1 Boehringer Ingelheim Ltd., Ellesfield Avenue, Bracknell, Berkshire UK; 2 Faculté de Pharmacie, University of Bordeaux, Bordeaux, France; 3 Respiratory Epidemiology, Occupational Medicine and Public Health, National Heart and Lung Institute, Imperial College London, London, UK

**Keywords:** Therapeutics, Chronic obstructive pulmonary disease, Diagnosis

## Abstract

We investigated the incidence of type II diabetes mellitus (T2DM) among people with COPD and whether exposure to inhaled corticosteroid (ICS) and exacerbation status was associated with T2DM. This descriptive cohort study used primary care data from the Clinical Practice Research Datalink (CPRD). The patient cohort included people with a diagnosis of COPD and previous smoking history registered at a CPRD practice between January 2010 and December 2016. We determined incidence rates by age, gender and deprivation. Using a nested case–control design—where cases and controls are drawn from the cohort population—we matched 1:5 with patients by age, gender and GP practice and estimated odds of T2DM using logistic regression (adjusting for smoking status, deprivation, BMI, hypertension, coronary heart disease and heart failure). We identified 220,971 COPD patients; mean age at COPD diagnosis was 66 years (SD 12) and 54% were male. The incidence rate of T2DM in COPD patients was 1.26 per 100 patient years (95% CI: 1.24–1.28) and was higher among men (1.32 vs 1.18 among women). The adjusted odds ratio for T2DM was 1.47 (95% CI: 1.36–1.60) among frequent exacerbators (≥2 treated exacerbations per year) compared to infrequent exacerbators and the odds ratio for patients receiving high-dose ICS (>800 mcg budesonide equivalent dose) was 1.73 (95% CI 1.65–1.82) compared to patients receiving no ICS therapy. Incidence of T2DM among COPD patients is high and exposure to ICS and frequent exacerbations are associated with a higher risk of T2DM among patients with COPD.

## Introduction

Chronic obstructive pulmonary disease (COPD) is the fourth leading cause of death in the world and is projected to become the third in 2020.^1^ According to the British Lung Foundation, an estimated 1.2 million people are living with diagnosed COPD in the UK, which translates to 4.5% of all people aged >40 years.

Despite its importance, COPD remains under-diagnosed and patients are often diagnosed late.^2^ Approximately 3 million people die globally of COPD each year.^3^

The presence of obstruction on spirometry has been found to be associated with diabetes^4^ and predictive of the development of diabetes.^5^ Mannino et al. found a modest association between patients with moderate COPD or higher and increased risk of diabetes. Similar findings were seen for lower-severity COPD.^6^

A study investigating factors associated with new-onset diabetes in the UK concluded that patients with frequent exacerbations of COPD and cardiac comorbidities, such as hypertension and heart disease, were more likely to develop type 2 diabetes mellitus (T2DM).^7^ Higher exposure of inhaled corticosteroid (ICS), a commonly prescribed treatment in COPD,^8^ was also associated with increased incidence of T2DM.^9^ However, a meta-analysis of the clinical trials with budesonide identified neither an increased risk of incident DM nor hyperglycaemia in patients with either asthma or with COPD.^10^

**Fig. 1:**
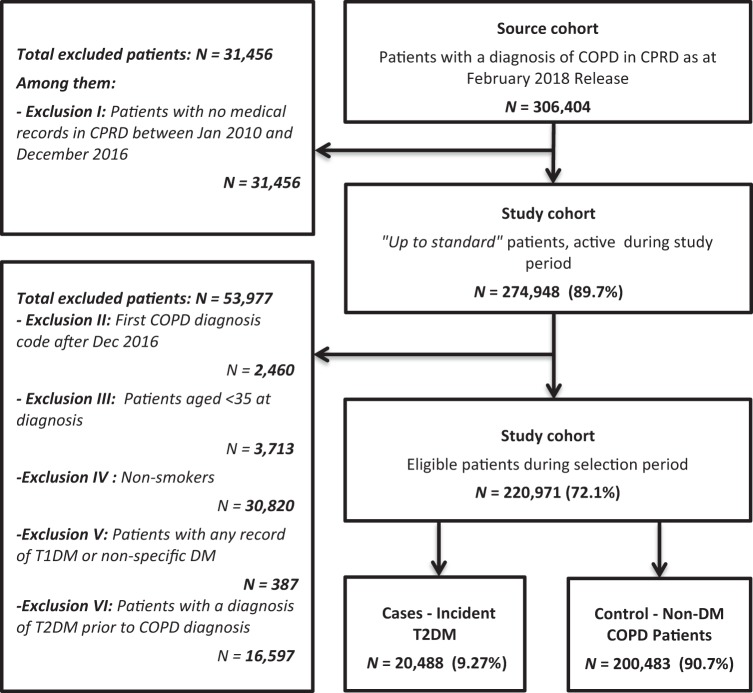
Flowchart of patients entering the study

As efficacy-driven randomised controlled trials are rarely powered to draw robust conclusions on safety endpoints, observational studies provide important data to further understanding of comorbid long-term conditions and their aetiologies. Heightened public concern over multi-morbidity provides stimulus to examine whether the previously observed association with diabetes remains contemporary.^11^ This study aims to calculate the prevalence and incidence of T2DM among COPD patients and investigate the risk factors associated with developing T2DM.

## Results

### Cohort analysis

A total of 220,971 COPD patients were identified during the study period (Fig. 1), with a mean age at diagnosis of 66 years (SD 12) and a proportion of 54% males. Among these patients, 20,488 cases (9.3%) reported T2DM, with an incidence rate of 1.26 per 100 patient-years (95% confidence interval (CI): 1.24–1.28). This was calculated with the number of cases out of person time in years (1,627,324).

This incidence was slightly higher among men than among women (1.32 vs 1.18 per 100 person years), and increased with age at COPD diagnosis until age 74 years. Incidence of T2DM among COPD patients was also higher for the most deprived areas (1.29 per 100 person-years) compared to the least deprived area (1.19 per 100 person-years) (Index of Multiple Deprivation (IMD) quintile). Similarly, cumulative prevalence of T2DM was higher in men (9.8%) than in women (8.7%) and ranged between 4.3% and 11.2% across the different age groups, with peak prevalence in age range 54–64 years (Table 1). Cumulative prevalence was defined as the number of new T2DM cases out of all diagnosed COPD patients.

**Table 1:** Prevalence and incidence of T2DM among COPD patients

	Incidence rate per 100 person years (95% CI)	Percentage cumulative prevalence (95% CI)
Overall	1.26 (1.24–1.28)	9.27 (9.15–9.39)
Gender		
Men	1.32 (1.30–1.35)	9.75 (9.58–9.92)
Women	1.18 (1.16–1.21)	8.71 (8.53–8.88)
Age (at COPD diagnosis), years		
<44	0.67 (0.62–0.72)	9.03 (8.45–9.61)
44–54	1.04 (1.00–1.08)	10.41 (10.07–10.75)
54–64	1.35 (1.32–1.38)	11.23 (10.98–11.48)
64–74	1.45 (1.41–1.48)	9.61 (9.38–19.83)
74–80	1.38 (1.32–1.44)	7.22 (6.92–7.51)
>80	1.19 (1.12–1.27)	4.33 (4.07–4.60)
IMD quintile		
1—least deprived	1.19 (1.15–1.24)	8.75 (8.43–9.06)
2	1.24 (1.21–1.28)	9.22 (8.95–9.49)
3	1.28 (1.24–1.32)	9.52 (9.25–9.78)
4	1.26 (1.22–1.30)	9.12 (8.84–9.40)
5—most deprived	1.29 (1.26–1.33)	9.51 (9.27–9.75)

*IMD* Index of Multiple Deprivation, *COPD* chronic obstructive pulmonary disease, *T2DM* type 2 diabetes mellitus, *CI* confidence interval

### Case–control analysis

For many variables including age at COPD diagnosis, duration of COPD, smoking status, body mass index (BMI) and cardiac comorbidities, results showed a significant association with T2DM incidence (*p* < .05; Table 2). Through a univariate analysis, we found that the odds ratio (OR) of diabetes incidence increased significantly over the BMI groups: 2.06 (95% CI: 1.72–2.47) for the 18.50–24.9 kg/m^2^ interval and 19.83 (95% CI: 15.90–24.74) for ≥40 kg/m^2^. Patients with moderate–severe COPD (GOLD stages 2 and 3) may be at increased risk of T2DM (Supplementary Table 4). However, no association was found when comparing quintiles of deprivation (IMD).

**Table 2:** Baseline characteristics of T2DM cases and their matched controls, before and after matching

Characteristics	Unmatched					Age–gender–practice matched						
	Cases, incident T2DM		Controls, non-DM		Standardised difference^a^	Cases, incident T2DM		Controls, non-DM		Standardised difference	Pre-match *T* test *p* value^b^	Post-match *T* test *p* value
	*N*	%	*N*	%		*N*	%	*N*	%			
*N*	20,488	9%	200,483	91%		19,841	24%	63,228	76%			
Male	11,652	57%	107,843	54%	0.06	11,286	57%	35,875	57%	0.00	<.0001	.72
Mean age at diagnosis	63.5 ± 10.5		65.8 ± 11.8		−0.20	61.6 ± 9.9		63.9 ± 10.2		0.23	<.0001	<.0001
Duration of COPD (years)	6.7 ± 6.5		7.4 ± 6.9		−0.12	6.19 ± 5.7		10.1 ± 8.1		−0.56	<.0001	<.0001
Age at COPD diagnosis												
<44	847	4%	8528	4%	0.27	628	3%	3235	5%	0.24	<.0001	<.0001
44–54	3216	16%	27,674	14%		2981	15%	11,628	18%			
54–64	6839	33%	54,075	27%		6715	34%	23,221	37%			
64–74	6415	31%	60,373	30%		6373	32%	19,016	30%			
74–80	2188	11%	28,133	14%		2177	11%	4847	8%			
>80	983	5%	21,700	11%		967	5%	1281	2%			
Smoking status												
Ex-smoker	9885	48%	98,135	49%	0.15	5330	27%	13,018	21%	0.15	<.0001	<.0001
Current smoker	6696	33%	78,119	39%		7101	36%	23,445	37%			
Missing	2895	14%	13,891	7%		7410	37%	26,765	42%			
BMI (kg/m^2^)												
<18.50	136	1%	7269	4%	0.53	164	1%	1694	3%	0.53	<.0001	<.0001
18.50–24.9	2278	11%	53,118	26%		2246	11%	14,038	22%			
25–29.9	4203	21%	38,879	19%		4166	21%	10,284	16%			
30–39.9	4116	20%	20,291	10%		4054	20%	4797	8%			
>40	571	3%	1781	1%		609	3%	363	1%			
Missing	9184	45%	79,145	39%		8602	43%	32,052	51%			
Hypertension	6228	30%	49,830	25%	0.12	6183	31%	11,879	19%	0.29	<.0001	<.0001
Coronary heart disease	2621	13%	18,862	9%	0.11	2605	13%	4729	7%	0.19	<.0001	<.0001
Heart failure	995	5%	9322	5%	0.01	987	5%	1476	2%	0.14	.1816	<.0001
IMD												
1—least deprived	2747	13%	28,665	14%	0.03	2640	13%	8243	13%	0.02	.0027	.0082
2	4115	20%	40,530	20%		3980	20%	12,311	19%			
3	4407	22%	41,902	21%		4266	22%	13,469	21%			
4	3711	18%	36,989	18%		3586	18%	11,583	18%			
5—most deprived	5508	27%	52,397	26%		5369	27%	17,622	28%			

*IMD* Index of Multiple Deprivation, *COPD* chronic obstructive pulmonary disease, *T2DM* type 2 diabetes mellitus, *BMI* body mass index

^a^Standardised differences calculated using Cohen’s *D* statistic

^b^*T* tests assess difference in empirical mean and *p* values test equality of pooled variances

Using a logistic regression model, association between ICS exposure, frequent exacerbations (two or more exacerbations per year) and T2DM comorbidity was calculated, adjusting for remaining non-matched confounders: smoking status, socioeconomic status, BMI, hypertension, coronary heart disease, heart failure, and COPD duration (Supplementary Table 2).

The risk of T2DM was greatest among frequent exacerbators compared to infrequent exacerbators (OR 1.47, 95% CI: 1.36–1.60). The adjusted OR for patients receiving ICS significantly increased with the higher doses, compared to patients receiving no ICS therapy: OR 1.73 (95% CI 1.65–1.82) for very high daily dose of ICS (>1600 mcg per day). Additionally, patients with a high number of ICS prescriptions were more likely to develop T2DM: OR 1.83 (95% CI: 1.48–2.26) for 16–20 prescriptions and 1.61 (95% CI 1.53–1.69) for 1–5 prescriptions (Supplementary Table 3, Table 3). No significant interactions were found between exacerbations and ICS use after adjusting for the known confounders.

**Table 3:** Sensitivity analysis without patients with asthma

Risk factors	T2DM (*N* = 11,456)	Non-DM (*N* = 37,433)	Adjusted OR (95% CI)
Frequent exacerbation	3%	2%	1.47 (1.29–1.67)
Inhaled corticosteroid (daily dose)^a^			
None	56%	66%	1
Low	10%	7%	1.47 (1.36–1.59)
Moderate	7%	6%	1.35 (1.24–1.48)
High	11%	9%	1.46 (1.36–1.58)
Very high	15%	12%	1.58 (1.47–1.68)
Number of prescriptions			
None	56%	56%	1
1–5	20%	16%	1.49 (1.40–1.59)
6–10	10%	12%	1.49 (1.39–1.60)
11–15	6%	8%	1.44 (1.33–1.57)
16–20	0%	0%	1.55 (1.09–2.20)
>20	7%	7%	1.48 (1.34–1.62)

*T2DM* type 2 diabetes mellitus, *OR* odds ratio, *CI* confidence interval

^a^Inhaled corticosteroid converted to budesonide equivalent dose. Daily dose (mcg): low (200–400), moderate (>400–800), high (>800–1600), very high (>1600)

### Sensitivity analyses

After excluding patients with comorbid asthma, there was still a significant association between frequent exacerbators and risk of developing T2DM: OR 1.47 (95% CI 1.29–1.67). The association also remained for the exposure of ICS, with a higher risk of developing T2DM among patients receiving a very high daily dose: OR 1.58 (95% CI: 1.47–1.68) (Table 3). Furthermore, after including interaction terms between ICS exposure and exacerbations on the risk of T2DM we found no relationship between exacerbations and ICS prescriptions (*p* > .1131 for all categories of ICS prescriptions) and between exacerbations and ICS dose (*p* > .3302 for all dose categories).

## Discussion

This study suggests that the incidence of T2DM among COPD patients is high, with a prevalence of 9% corresponding to 111,240 cases out of the 1.2 million COPD patients in the UK. Comparatively, Zghebi et al. found a prevalence of T2DM of 5.3% (5.2; 5.23) in 2014 over the UK population.^12^

Although the mechanisms explaining the relationship between respiratory impairment and diabetes remain unclear, Lin et al. suggested that systemic inflammation could be a plausible explanation.^13^ In patients with COPD, the levels of inflammatory mediators (such as tumour necrosis factor alpha, interleukin-6 or C-reactive protein) were showed to be increased. According to the same study, these mediators may contribute to the development of type 2 diabetes.

Additionally, our study showed that other factors could contribute to the onset of T2DM: after adjustment for personal risk factors, we found that exposure to high doses of ICS and frequent exacerbations of COPD are associated with a higher risk of developing T2DM among patients with COPD. According to Suissa et al.,^14^ they could also have an impact on diabetes progression. The contribution of these factors on the incidence of T2DM is a relevant finding, when ICS are known to be commonly prescribed for frequent exacerbators of COPD^11^.

Moreover, results show that the risk of T2DM is high irrespective of ICS doses. This contrasts previous research by Suissa et. al who found a dose–response effect of ICS use on both the incidence and progression of diabetes among COPD and asthma patients in Quebec.^14^ Our results suggest the risk is elevated irrespective of dose; however, further research is warranted among a UK cohort.

Patients with cardiac comorbidities and high BMI were found to be associated with a higher risk of developing T2DM. In a previous study, Ho et al. showed that the onset of T2DM could even worsen outcomes for these patients and have an impact on mortality among the COPD population.^7^

The significant factors identified in this study are those that can be identified and modified, but this requires the contribution of both patients and health-care providers in clinical care of COPD.

Our findings are consistent with a previous study, based on the association between ICS and diabetes incidence in COPD population.^14^ Even if slight differences exist with our study, such as the size of the population or the time of follow-up, the main findings were similar.

However, a previous study of all the clinical trials of the ICS budesonide did not identify any increased risk of new-onset DM in patients with COPD.^10^ This contrast with our study results could be explained by the choice of their population, who were for the most part comorbidity free, and the length of follow-up, which could have underestimated the effect of exposure to ICS on incidence of diabetes.

While Clinical Practice Research Datalink (CPRD) strives to provide high-quality data, there are some limitations to the analysis. Indeed, the quality of the data set relies on correct data entry by health practitioners; therefore it is possible that there may be missing values where data have not been recorded. In addition, we were not able to match 1:5 cases to controls; however, this did not affect the precision of the results, and our sample size is generalisable to the COPD population. Prescription data are likely to be entered correctly as the electronic system requires that the data are input in order to generate a patient prescription—however, as it is the case with this type of database, we cannot know if the prescription was dispensed or whether the patient took the medication.

In addition, there are number of variables that are not currently measured well in electronic data (e.g. physical activity, alcohol consumption, diet) that are related to the incidence of diabetes. These may affect the development of disease and therefore conclusions from this study should be interpreted with caution as there are no adequate proxy measures for these within the data, and though our algorithm for identification of patients with T2DM utilised several variables in addition to diagnostic codes, such as treatment and age at diagnosis, there still remains a risk of some misclassification of T2DM.

As highlighted in this study, COPD patients with frequent exacerbations are more likely to develop T2DM. Similarly, high exposure to ICS, which is the most commonly used treatment for these patients, showed a significant association with the onset of diabetes. In this perspective, it would be interesting to re-examine the risk–benefit profile of this treatment in COPD clinical care and limit the use of high-dose ICS to patients where the benefit is clear.

Moreover, targeted surveillance should be set up in the future for COPD patients with cardiac comorbidities and high BMI, as they are also more likely to develop diabetes. Such management in COPD clinical care suggests that some changes may be needed in the next COPD guidelines, in order to control and try to modify these risk factors.

The mechanisms underlying the association between ICS exposure and incidence of T2DM has not been fully elucidated and warrants further investigation.

This study suggests that a significant proportion of the COPD population is at high risk of developing T2DM due to many risk factors, including exposure to ICS and frequency of exacerbations. Therefore, when assessing the risk benefit of high-dose ICS in individual COPD patients, it is important to consider the possibility of increased incidence of diabetes.

## Methods

### Study design

A nested case–control study was conducted in a retrospective cohort of 220,971 subjects selected from the UK CPRD from January 2010 to December 2016. The CPRD provides a database of anonymised longitudinal clinical records from general practice, covering 689 GP practices in the UK, with 3.9 million people available for observation at the start of the study. The geographical distribution is representative of the UK population,^15^ and several studies have confirmed the high quality of the data and completeness of the clinical records.^16–20^ As such, it is recognised as a reliable source for investigating UK general practice and prescribing and has been used in many previous studies related to COPD.^21,22^

### Population

Patients of interest for the cohort were those with a coded diagnosis of COPD (see Supplementary Table 1) prior to the study registered at a CPRD practice during the study period (January 2010 to December 2016). Patients also had to be aged ≥35 years at the date of diagnosis and be current or former smokers, anytime up to COPD diagnosis. Indeed, smoking is known to be a dominating risk factor for COPD: a previous study showed that the proportion of ever smokers among subjects with COPD was 77%.^23^

The follow-up of the cohort started from the diagnosis of COPD and ended whether with a loss to follow-up, patient’s death or patient’s diagnosis of T2DM.

As the objective was to study the prevalence and incidence of T2DM in COPD patients, we excluded the subjects with a diagnosis of T2DM prior to COPD diagnosis. Patients with any records of T1DM or non-specific DM were also excluded.

20,488 of T2DM were detected during the study period as the case group, and 200,483 non-T2DM COPD patients were selected from the cohort as controls. In order to control for known confounding factors, cases were matched to controls in a ratio of 1:5 by age at COPD diagnosis (5 years band), gender and within GP practice.

### Analysis

Baseline data (date of COPD diagnosis) were analysed descriptively among the cohort population. Variables of interest, including duration of COPD, age, gender, smoking status, BMI, cardiac comorbidities, and social deprivation, were compared between the T2DM and non-T2DM patient groups.

Univariate analysis was carried out among matched cases and controls to identify the variables that were significantly associated with the incidence of T2DM (using logistic regression). A final multivariate logistic regression model was used to investigate factors associated with the incidence of T2DM. This included: the number of ICS prescriptions per person-year or the latest prescribed dose [calculated as budesonide equivalent: Daily dose (mcg): low (200–400), moderate (>400–800), high (>800–1600), very high (>1600)], the number of exacerbations per person-year [defined using an adapted version of a validated algorithm for use in CPRD data^24^ as antibiotic and oral corticosteroid prescriptions for 5–14 days were calculated using the date of prescription and drug pack information or coded lower respiratory tract infection or coded acute exacerbation], smoking status, BMI, hypertension, coronary heart disease, heart failure, deprivation, and duration of COPD.

Data were extracted using the online version of CPRD (CPRD-GOLD) and analysed using the SAS software version 9.4. Missing data that occurred in covariates or descriptive variables were classified as missing; no imputation was conducted.

### Ethical approval

This study was reviewed and approved by the Independent Scientific Advisory Committee (ISAC) for Medicines and Healthcare products Regulatory Agency (MHRA) database research (Ref: 15_237R) and an internal scientific committee within the study sponsor.

### Reporting summary

Further information on experimental design is available in the Nature Research Reporting Summary linked to this article.

## Supplementary information

Supplementary Information

Reporting Summary

## Data Availability

Data are available on request from the CPRD. Their provision requires the purchase of a license, and our license does not permit us to make them publicly available to all. We used data from the version collected in February 2018 and have clearly specified the data selected in our “Methods” section. To allow identical data to be obtained by others, via the purchase of a license, we will provide the code lists on request. Licences are available from the CPRD (http://www.cprd.com): The Clinical Practice Research Datalink Group, The Medicines and Healthcare products Regulatory Agency, 5th Floor, 151 Buckingham Palace Road, Victoria, London SW1 W 9SZ, UK.

## Supplementary information

**Supplementary Information** accompanies the paper on the *npj Primary Care Respiratory Medicine* website (10.1038/s41533-019-0138-6).
