# Supplementary material for: Incidence of type II diabetes in chronic obstructive pulmonary disease: a nested case–control study
Source: NPJ Prim Care Respir Med. 2019 Jul 15;29:28. doi: 10.1038/s41533-019-0138-6 (PMC6629671; doi:10.1038/s41533-019-0138-6)
Supplement: Supplementary file 1 — Supplementary Information [file 41533_2019_138_MOESM1_ESM.docx]

**Supplementary Information**

**S1 COPD codes**

| **medical code** | **read code** | **read term** |
| --- | --- | --- |
| 794 | H32..00 | Emphysema |
| 998 | H3...11 | Chronic obstructive airways disease |
| 1001 | H3...00 | Chronic obstructive pulmonary disease |
| 3243 | H31..00 | Chronic bronchitis |
| 5710 | H3z..00 | Chronic obstructive airways disease NOS |
| 9876 | H38..00 | Severe chronic obstructive pulmonary disease |
| 10802 | H37..00 | Moderate chronic obstructive pulmonary disease |
| 10863 | H36..00 | Mild chronic obstructive pulmonary disease |
| 11287 | 66YM.00 | Chronic obstructive pulmonary disease annual review |
| 12166 | H3y..00 | Other specified chronic obstructive airways disease |
| 14798 | H312100 | Emphysematous bronchitis |
| 15157 | H31x.00 | Chronic bronchitis NOS |
| 15782 | G41z.00 | Chronic pulmonary heart disease NOS |
| 16410 | H32yz00 | Other emphysema NOS |
| 21061 | H3y0.00 | Chronic obstruct pulmonary dis with acute lower resp infectn |
| 23492 | H320z00 | Chronic bullous emphysema NOS |
| 25603 | H310.00 | Simple chronic bronchitis |
| 26082 | H541000 | Chronic pulmonary oedema |
| 26306 | H320.00 | Chronic bullous emphysema |
| 27819 | H312.00 | Obstructive chronic bronchitis |
| 33450 | H32z.00 | Emphysema NOS |
| 37247 | H3z..11 | Chronic obstructive pulmonary disease NOS |
| 40788 | H32y.00 | Other emphysema |
| 44525 | H312z00 | Obstructive chronic bronchitis NOS |
| 45770 | 66Yg.00 | Chronic obstructive pulmonary disease disturbs sleep |
| 45771 | 66Yh.00 | Chronic obstructive pulmonary disease does not disturb sleep |
| 46578 | H321.00 | Panlobular emphysema |
| 54893 | H582.00 | Compensatory emphysema |
| 56860 | H320000 | Segmental bullous emphysema |
| 60188 | H320200 | Giant bullous emphysema |
| 61118 | H310z00 | Simple chronic bronchitis NOS |
| 64721 | H464000 | Chronic emphysema due to chemical fumes |
| 65733 | Hyu3100 | [X]Other specified chronic obstructive pulmonary disease |
| 66043 | H31y.00 | Other chronic bronchitis |
| 66058 | Hyu3000 | [X]Other emphysema |
| 67040 | H3y..11 | Other specified chronic obstructive pulmonary disease |
| 68066 | H31yz00 | Other chronic bronchitis NOS |
| 68662 | H320100 | Zonal bullous emphysema |
| 70787 | H32y100 | Atrophic (senile) emphysema |
| 93568 | H39..00 | Very severe chronic obstructive pulmonary disease |
| 99536 | H320300 | Bullous emphysema with collapse |

# S2 Adjusted OR of frequent exacerbators as a risk factor for developing T2DM in COPD population

|  | |  | |  | |  | |  | |  | |
| --- | --- | --- | --- | --- | --- | --- | --- | --- | --- | --- | --- |
| **Characteristics** | **Incident T2DM** | | | | **Non-DM** | | | | **OR (95% CI)** | |  |
|  | N | | % | | N | | % | |  |  |  |
| Frequent Exacerbation | 995 | | 5% | | 2,183 | | 3% | | 1.47 (1.36 - 1.60) | |  |
|  |  | |  | |  | |  | |  | |  |
| Smoking Status |  | |  | |  | |  | |  | |  |
| Ex Smoker | 5,330 | | 27% | | 13,018 | | 21% | | 1 | |  |
| Current smoker | 7,101 | | 36% | | 23,445 | | 37% | | 1.02 (0.97 - 1.07) | |  |
|  |  | |  | |  | |  | |  | |  |
| BMI (kg/m2) |  | |  | |  | |  | |  | |  |
| Underweight | 164 | | 1% | | 1,694 | | 3% | | 1 | |  |
| Normal Weight | 2,246 | | 11% | | 14,038 | | 22% | | 2.05 (1.70 - 2.46) | |  |
| Overweight | 4,166 | | 21% | | 10,284 | | 16% | | 4.76 (3.96 - 5.71) | |  |
| Obese | 4,054 | | 20% | | 4,797 | | 8% | | 9.22 (7.67 - 11.08) | |  |
| Severely Obese | 609 | | 3% | | 363 | | 1% | | 15.65 (12.51 - 19.58) | |  |
|  |  | |  | |  | |  | |  | |  |
| Hypertension | 6,183 | | 31% | | 11,879 | | 19% | | 1.33 (1.28 - 1.39) | |  |
|  |  | |  | |  | |  | |  | |  |
| Chronic Heart Disease | 2,605 | | 13% | | 4,729 | | 7% | | 1.37 (1.30 - 1.45) | |  |
|  |  | |  | |  | |  | |  | |  |
| Heart Failure | 987 | | 5% | | 1,476 | | 2% | | 1.34 (1.22 - 1.46) | |  |
|  |  | |  | |  | |  | |  | |  |
| IMD |  | |  | |  | |  | |  | |  |
| 1 - least deprived | 2,640 | | 13% | | 8,243 | | 13% | | 1 | |  |
| 2 | 3,980 | | 20% | | 12,311 | | 19% | | 1.00 (0.94 - 1.06) | |  |
| 3 | 4,266 | | 22% | | 13,469 | | 21% | | 1.00 (0.94 - 1.06) | |  |
| 4 | 3,586 | | 18% | | 11,583 | | 18% | | 0.86 (0.91 - 1.02) | |  |
| 5 - most deprived | 5,369 | | 27% | | 17,622 | | 28% | | 0.96 (0.91 - 1.02) | |  |
|  |  | |  | |  | |  | |  | |  |
| COPD duration [mean, SD] | 6.19 ± 5.7 | |  | | 10.1 ± 8.1 | |  | | 0.92 (0.91 - 0.92) | |  |

Frequent exacerbation defined as the number of exacerbations over a year. T2DM: Type 2 Diabetes Mellitus, OR: Odds Ratio, CI: Confidence Interval, IMD: Index of Multiple Deprivation, BMI: Body Mass Index, COPD: Chronic Obstructive Pulmonary Disease. Chronic Heart Disease includes: coronary heart disease, ischaemic heart disease and, myocardial infarction.

**S3 Adjusted OR of ICS dose exposure for developing T2DM in COPD population**

| **Characteristics** | **Incident T2DM** | | **Non-DM** | | **OR (95% CI)** |
| --- | --- | --- | --- | --- | --- |
|  | N | % | N | % |  |
| Inhaled Corticosteroids (daily dose) | |  |  |  |  |
| None | 8,921 | 45% | 36,474 | 58% | 1 |
| Low | 2,351 | 12% | 5,395 | 9% | 1.60 (1.51 - 1.69) |
| Moderate | 1,825 | 9% | 4,738 | 7% | 1.51 (1.41 - 1.60) |
| High | 2,808 | 14% | 7,000 | 11% | 1.57 (1.49 - 1.66) |
| Very High | 3,936 | 20% | 9,621 | 15% | 1.73 (1.65 - 1.82) |
|  |  |  |  |  |  |
| Smoking Status |  |  |  |  |  |
| Ex-Smoker | 5,330 | 27% | 13,018 | 21% | 1 |
| Current smoker | 7,101 | 36% | 23,445 | 37% | 1.05 (1.00 - 1.10) |
|  |  |  |  |  |  |
| BMI (kg/m2) |  |  |  |  |  |
| Underweight | 164 | 1% | 1,694 | 3% | 1 |
| Normal Weight | 2,246 | 11% | 14,038 | 22% | 2.00 (1.67 - 2.41) |
| Overweight | 4,166 | 21% | 10,284 | 16% | 4.62 (3.85 - 5.55) |
| Obese | 4,054 | 20% | 4,797 | 8% | 8.98 (7.47 - 10.80) |
| Severely Obese | 609 | 3% | 363 | 1% | 15.12 (12.08 - 18.94) |
|  |  |  |  |  |  |
| Hypertension | 6,183 | 31% | 11,879 | 19% | 1.34 (1.29 - 1.40) |
|  |  |  |  |  |  |
| Chronic Heart Disease | 2,605 | 13% | 4,729 | 7% | 1.40 (1.33 - 1.48) |
|  |  |  |  |  |  |
| Heart Failure | 987 | 5% | 1,476 | 2% | 1.38 (1.26 - 1.51) |
|  |  |  |  |  |  |
| IMD |  |  |  |  |  |
| 1 - least deprived | 2,640 | 13% | 8,243 | 13% | 1 |
| 2 | 3,980 | 20% | 12,311 | 19% | 0.99 (0.93 - 1.05) |
| 3 | 4,266 | 22% | 13,469 | 21% | 0.99 (0.93 - 1.05) |
| 4 | 3,586 | 18% | 11,583 | 18% | 0.96 (0.90 - 1.02) |
| 5 - most deprived | 5,369 | 27% | 17,622 | 28% | 0.96 (0.91 - 1.02) |
|  |  |  |  |  |  |
| COPD duration [mean, SD] | 6.19 ± 5.7 |  | 10.1 ± 8.1 |  | 0.92 (0.91 - 0.92) |

Inhaled corticosteroids converted to budesonide equivalent dose. Daily dose(µg) : low (200-400), moderate (>400-800), high (>800-1600), very high (>1600). T2DM: Type 2 Diabetes Mellitus, OR: Odds Ratio, CI: Confidence Interval, IMD: Index of Multiple Deprivation, BMI: Body Mass Index, COPD: Chronic Obstructive Pulmonary Disease. Chronic Heart Disease includes: coronary heart disease, ischaemic heart disease and, myocardial infarction.

**S4 Adjusted OR of GOLD Stage for developing T2DM in COPD population**

| **Characteristics** | **Incident T2DM** | | **Non-DM** | | **OR (95% CI)** |
| --- | --- | --- | --- | --- | --- |
|  | N | % | N | % |  |
| GOLD Stage FEV_1_% predicted | |  |  |  |  |
| 1 (>80) | 1272 | 6% | 4473 | 7% | 1 |
| 2 (50-79) | 5266 | 27% | 14658 | 23% | 1.265 (1.175 - 1.361) |
| 3 (30-49) | 2953 | 15% | 9567 | 15% | 1.325 (1.224 - 1.433) |
| 4 (<30) | 696 | 4% | 3945 | 6% | 0.952 (0.856 - 1.060) |
|  |  |  |  |  |  |
| Smoking Status |  |  |  |  |  |
| Ex-Smoker | 3781 | 19% | 9444 | 15% | 1 |
| Current smoker | 4698 | 24% | 15802 | 25% | 1.03 (0.98 - 1.09) |
|  |  |  |  |  |  |
| BMI (kg/m2) |  |  |  |  |  |
| Underweight | 82 | 0% | 1122 | 2% | 1 |
| Normal Weight | 1605 | 8% | 9913 | 16% | 2.17 (1.72 - 2.78) |
| Overweight | 2929 | 15% | 7578 | 12% | 4.76 (3.78 - 6.00) |
| Obese | 2896 | 15% | 3645 | 6% | 9.05 (7.18 - 11.41) |
| Severely Obese | 386 | 2% | 274 | 0% | 15.05 (11.42 - 19.83) |
|  |  |  |  |  |  |
| Hypertension | 3797 | 19% | 7446 | 12% | 1.36 (1.29 - 1.43) |
|  |  |  |  |  |  |
| Chronic Heart Disease | 1417 | 7% | 2505 | 4% | 0.42 (1.32 - 1.53) |
|  |  |  |  |  |  |
| Heart Failure | 420 | 2% | 606 | 1% | 1.33 (1.16 - 1.52) |
|  |  |  |  |  |  |
| IMD |  |  |  |  |  |
| 1 - least deprived | 1370 | 7% | 4368 | 7% | 1 |
| 2 | 2064 | 10% | 6481 | 10% | 1.01 (0.93 - 1.10) |
| 3 | 2214 | 11% | 7147 | 11% | 0.99 (0.92 - 1.08) |
| 4 | 1804 | 9% | 5882 | 9% | 0.98 (0.90 - 1.07) |
| 5 - most deprived | 2735 | 14% | 8765 | 14% | 1.01 (0.94 - 1.10) |
|  |  |  |  |  |  |
| COPD duration [mean, SD] | 6.12 ± 5.3 |  | 8.77 ± 6.77 |  | 0.94 (0.93 - 0.94) |

Figures represent the cohort of patients with available lung function data prior to T2DM (N =42,830). Number of ICS prescriptions over a year. T2DM: Type 2 Diabetes Mellitus, OR: Odds Ratio, CI: Confidence Interval, IMD: Index of Multiple Deprivation, BMI: Body Mass Index, COPD: Chronic Obstructive Pulmonary Disease. Chronic Heart Disease includes: coronary heart disease, ischaemic heart disease and, myocardial infarction.
